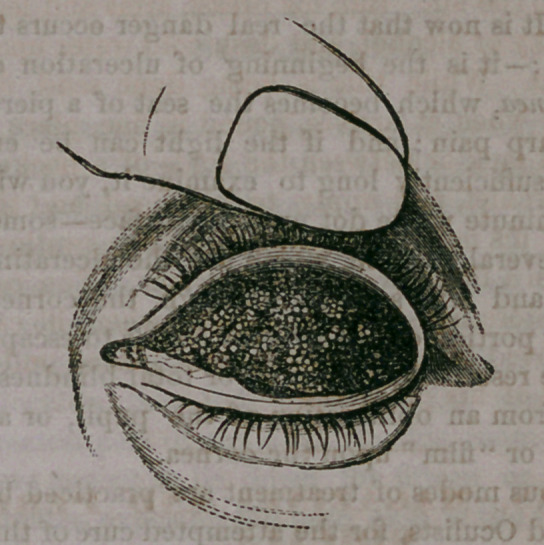# Granular Lids

**Published:** 1870-07

**Authors:** 


### The Bistoury.

ELMIRA, N. Y., JULY, 1870.

The BISTOURY is published Quarterly, upon the 1st of April, July, October and January, at Fifty Cents a year in advance.

Club Rates :--$25, will be paid for every 100 subscribers.

### GRANULAR LIDS.

In the nomenclature of diseases of the eye, there is probably no term more familiar to the general reader than that of the "granular lid;" yet no disease of the eye is so little understood and so badly managed.

We believe we are correct in saying that nearly two-thirds of the cases of blindness that may be found in our Blind Asylums, are caused directly, by the granular lid, assisted in the majority of cases by bad management and worse treatment. The granular lid is generally caused by the irritation of foreign bodies coming in contact with the eye—such as small seeds, sand, dust, and other substances, continually to be found floating in the air. These particles find a lodgment under the lid, where they at first produce a slight smarting or itching sensation, causing the patient to rub the eyes vigorously with the fingers or handkerchief. This process only tends to imbed the foreign particles more firmly in the conjunctiva, (covering membrane of the eye and lids,) where it creates additional inflammation and the formation of the granulations. At this stage of the disease, the eye appears red and inflamed—the lids are puffed up, swollen, and hot, scalding tears continually escape upon the cheek, often excoriating the skin, from the surface of which little pustules are seen to arise. If you are skilled in everting the eye lid, and will examine its under surface, you will find it completely covered with granulations—a fungus growth, resembling the spawn of small fish. Sometimes they will be distributed over the lid in small groups; again they will be piled up, one upon the other, until the mass becomes half an inch in thickness. At such times they are found to be very much injected with blood, and will bleed freely upon the slightest touch. Again, they will assume a paler hue, and will be more firm to the touch—often quite cartilaginous, causing much more violent irritation to the eye, and resulting much sooner in entire destruction of the organ. Below we give a very accurate illustration of granulations upon the upper lid. The lid is represented as everted, exposing the groups of fungus growths upon the under surface.

This disease is also found in the lower lids, but is more frequently confined to the upper. In cases of long duration both upper and lower lids are equally diseased. These granulations cause as much irritation to the eye as would so much sand, were it spread upon the under surface of the lid. The eye becomes congested and inflamed—vessels shoot over the *cornea* in every possible direction, forming so dense a net-work as to almost entirely exclude the light. Great quantities of matter are generally discharged—particularly at night, the patient finding the eye-lids glued together, and feeling much as though thrust full of sticks. Considerable bathing of the lids is necessary before the patient can separate them, or allow the light to reach the eye. Often sneezing is occasioned as soon as the eyes are fairly opened; at such times very little or no light at all can be endured, and the lids become very painful to the touch—the swelling is increased, as well as the discharge of matter.

At this stage, the disease becomes contagious, if the matter comes in contact with the eyes of another person. Patients should therefore be required to use none but their own towels, soap and basin; and a continual watch should be kept over them, to see that they adhere strictly to such instructions. All articles of clothing used by them should be kept out of the reach of children, for we have frequently seen an entire family inoculated with the disease, simply from the playful use of a patient's pocket-handkerchief, in the hands of a child.

These severe inflammatory symptoms continue during a period of from one to three months, when they gradually subside, the patient supposing that he is certainly getting well; when suddenly, without any apparent cause, he has another attack of inflammation and consequent pain, even more acute than his former attack. It is now that the real danger occurs to the eye;—it is the beginning of ulceration of the *cornea*, which becomes the seat of a piercing, sharp pain; and if the light can be endured sufficiently long to examine it, you will find a minute white dot upon its surface—sometimes several of them, which are the ulcerating points, and will soon eat through the cornea, allow a portion of the *aqueous humor* to escape, and the result will be partial or total blindness, either from an obliteration of the pupil, or an opacity or "film" upon the cornea.

Various modes of treatment are practiced by so-called Oculists, for the attempted cure of this troublesome difficulty of the lids. Some cauterize the granulations with nitrate of silver,—many with sulphate of copper, ("blue-stone,") —while others have secret remedies, washes and lotions, with which they torture their luckless victims. All applications that burn, smart and inflame the eye, as will the "remedies" just mentioned, are injurious; for, while they may subdue the granulations during the time that they are being constantly applied, their severity is doing an irreparable injury to the cornea,—causing the same train of results—and in much less time—that would happen from the ordinary course of the disease itself.

Many patients attempt to treat themselves with the numerous "eye-waters" that are advertised for sale; some try to "cleanse their blood" with various syrups, recommended by their neighbors, or by some "Indian Root Doctor;" others resort to physic, blistering, cupping, leeching, poulticing, and the like,—all to no purpose. Nothing but the absolute removal of the granulations will give relief; and all the plans resorted to, and to which we have just referred, only tend to aggravate and complicate the difficulty. We honestly believe that more cases of the granular lid are rendered hopelessly blind by such abominable applications as the above, than ever occurred through the natural course of the disease.

We therefore warn the subjects of this disease to refrain from the use of the dangerous nostrums that will be recommended to them by their anxious friends or the traveling quack, for they can do no possible good, but are almost certain to enhance the difficulty, and render a case that might have been restored to useful vision, under the care of a judicious and skillful surgeon, hopelessly and incurably blind. Don't try to "doctor" yourself in such cases—it is dangerous economy. Better apply to a regularly educated physician, and if possible, to one who makes the treatment of such cases his special practice;—avoid "Indian Doctors," traveling humbugs, and patent medicines—you will then have the satisfaction of knowing that you did nothing to hasten your own blindness.

## Figures and Tables

**Figure f1:**